# Synthesis of high quality 2D carbide MXene flakes using a highly purified MAX precursor for ink applications†

**DOI:** 10.1039/d0na00398k

**Published:** 2020-11-23

**Authors:** Shi-Hyun Seok, Seungjun Choo, Jinsung Kwak, Hyejin Ju, Ju-Hyoung Han, Woo-Seok Kang, Joonsik Lee, Se-Yang Kim, Do Hee Lee, Jungsoo Lee, Jaewon Wang, Seunguk Song, Wook Jo, Byung Mun Jung, Han Gi Chae, Jae Sung Son, Soon-Yong Kwon

**Affiliations:** Department of Materials Science and Engineering, Center for Future Semiconductor Technology (FUST), Ulsan National Institute of Science and Technology (UNIST) Ulsan 44919 Korea hgchae@unist.ac.kr jsson@unist.ac.kr sykwon@unist.ac.kr; Composites Research Division, Korea Institute of Materials Science (KIMS) Changwon 51508 Korea

## Abstract

The practical application of 2D MXenes in electronic and energy fields has been hindered by the severe variation in the quality of MXene products depending on the parent MAX phases, manufacturing techniques, and preparation parameters. In particular, their synthesis has been impeded by the lack of studies reporting the synthesis of high-quality parent MAX phases. In addition, controllable and uniform deposition of 2D MXenes on various large-scale substrates is urgently required to use them practically. Herein, a method of pelletizing raw materials could synthesize a stoichiometric Ti_3_AlC_2_ MAX phase with high yield and processability, and fewer impurities. The Ti_3_AlC_2_ could be exfoliated into 1–2-atom-thick 2D Ti_3_C_2_T_*x*_ flakes, and their applicability was confirmed by the deposition and additional alignment of the 2D flakes with tunable thickness and electrical properties. Moreover, a practical MXene ink was fabricated with rheological characterization. MXene ink exhibited much better thickness uniformity while retaining excellent electrical performances (*e.g.*, sheet resistance, electromagnetic interference shielding ability) as those of a film produced by vacuum filtration. The direct functional integration of MXenes on various substrates is expected to initiate new and unexpected MXene-based applications.

## Abstract 2

A method of pelletizing raw materials was used to tackle unwarranted variations in MXene products depending on the parent MAX phases, manufacturing techniques, and preparation parameters, enabling a direct painting process on various surfaces for ink applications.

## Graphical abstract

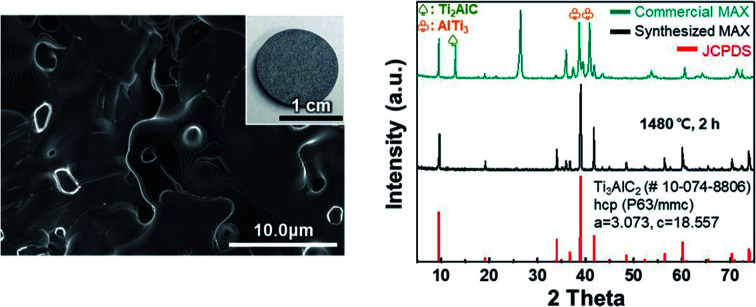

## 1. Introduction

The past few years have witnessed significant development in two-dimensional (2D) MXene research (*i.e.*, a large group of transition metal (TM) carbides or nitrides) as a new material for various applications owing to its combination of unique properties and 2D layered claylike structure.^1–3^ It is produced by the selective etching of A layers from their parent MAX phase (*i.e.*, precursor), where M is an early TM, A is generally an element of group IIIA or IVA (*i.e.*, group 13 or 14), and X is carbon and/or nitrogen.^4^ The structure of MXene has the general formula of M_*n*+1_X_*n*_T_*x*_ (*n* = 1–3) with M_*n*+1_X_*n*_ layers and surface-terminating functional groups represented by T_*x*_ (–OH, –F, –O, –H, *etc.*).^5^ Because of their high metallic conductivities^6^ and abundant surface functional groups that provide electrochemically active sites,^7^ MXenes have shown promising performance in energy storage devices, electromagnetic interference (EMI) shielding, catalysis, sensors, and many other applications.^8–13^ Above all, along with these advantages, the versatile chemistry and structural variety of MXenes allow us to tune the properties and make them competitive with other 2D materials.^14–17^ For instance, Ti_3_C_2_T_*x*_ MXene, the most-studied material in the family to date, has been exfoliated from the precursor Ti_3_AlC_2_ phase prepared by pressureless sintering and has shown promise in numerous applications.^18–20^ However, compared to the reports on the applications of MXenes, there are relatively fewer recent reports on the synthesis, manufacturing techniques, and preparation parameters of their precursor MAX phases,^21,22^ which decisively affects the quality and composition of the final MXenes.^23,24^ Moreover, the effect of sintering conditions for the MAX phase on different impurities and the resulting yield for the exfoliation of MXene is yet to be studied. Pressure-assisted sintering methods for the preparation of dense bulk MAX phases, such as hot isostatic pressing,^21^ hot pressing,^22^ spark plasma sintering,^25^ and self-propagating high-temperature synthesis,^26^ have the disadvantages of high cost and complex processes. On the other hand, commercially available MAX powders are often contaminated to some extent with intermediate phases and other impurities, in which the non-stoichiometric composition leads to the reduction of desired phases and decreased crystallinities of MXenes.^27,28^ Collectively, improving the quality control of parent MAX phases as well as in-depth study of the preparation parameters and processing equipment, which strongly influence the structural features of the final MXenes, are critical for mass production.

In addition to synthesis challenges for high-quality MXenes, efforts to obtain MXene-based membranes and coatings through various methods and to incorporate them into hosts are highly required. Very recently, as a method to utilize MXene obtained from the precursor MAX phase, developments of viscous aqueous inks have been reported.^29–31^ MXene inks, which were made of MXene flakes and had hydrophilicity and a high surface charge, could be easily applied on a variety of substrates using affordable strategies such as writing, printing, stamping, and painting. However, MXene inks reported to date are rarely composed of only delaminated-MXene flakes (*i.e.*, frequent presence of bulky structure in deposited flakes), and the films made from the inks do not exhibit laterally stacked morphologies.^29–31^ The uneven stacking of MXene flakes could lead to a reduction in conductivity, and the applications of the films are also limited to electrical circuits or electrodes for energy storage devices. Therefore, formulating an MXene ink with ideal viscoelastic properties, which would allow the 2D flakes to be deposited more uniformly and laterally, is highly necessary at this moment.

Herein, raw material powders were pelletized for a uniform and dense sintering reaction, leading to a synthetic Ti_3_AlC_2_ MAX phase with a high yield and processability featured by much fewer impurities even though it was simpler than conventional ways with fewer process variables. We further optimized the sintering process by observing phase changes in the pellet based on various structural analyses, which resulted in the formation of an ideal Ti_3_AlC_2_ MAX phase pellet with high crystallinity and accurate stoichiometry in comparison to the commercially available MAX powders. The synthesized Ti_3_AlC_2_ could be exfoliated into 1–2-atom-thick 2D flakes of Ti_3_C_2_T_*x*_ MXene, resulting in high-concentration dispersion in DI water with high purity. The vacuum filtration method (followed by a hot-pressing process) allows the uniform and controllable deposition of MXene membranes with thicknesses ranging from several layers to tens of micrometers over large areas. The electronic properties can thus be tuned over three orders of magnitude, making them potentially useful for various flexible devices. The effectiveness of EMI shielding, which is one of the promising applications using MXenes, can also be effectively tuned, confirming their applicability. Moreover, a practical MXene ink that can be easily painted on various substrates was fabricated *via* a process to concentrate pure delaminated MXene flakes. Rheological properties of the MXene ink were investigated to optimize painting conditions so that the 2D flakes could be deposited with a uniform thickness and conductivity on substrates of various surfaces. Notably, painted MXene ink exhibited much better thickness uniformity while keeping a similar morphology and electrical properties to those of a film produced by vacuum filtration, thus showing the possibility for more diverse applications. Finally, excellent EMI shielding ability in both filtrated and painted MXene films without any additives was achieved, which is comparable with pure aluminum and copper foils.

## 2. Results and discussion

**Fig. 1:**
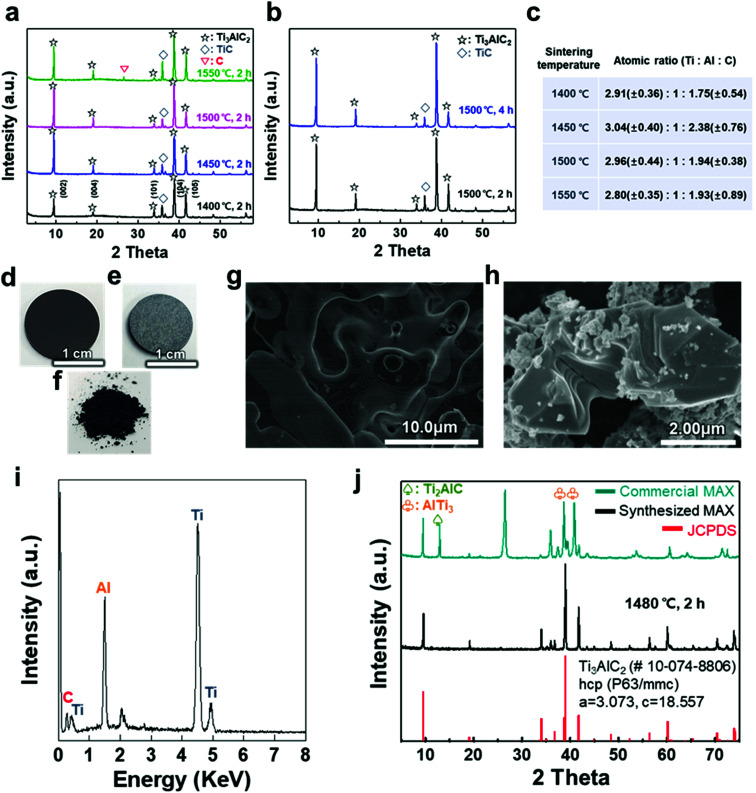
(a) XRD patterns of as-milled Ti_3_AlC_2_ MAX phase pellets sintered at various temperatures, (b) XRD patterns of the pellets sintered at 1500 °C at different times, and (c) calculated atomic ratio based on sintering temperature. Photographs of a cold-pressed pellet (d) before and (e) after the sintering process at ≈1480 °C for 2 h, and (f) as-milled powder. SEM images of the (g) sintered pellet surface and (h) as-synthesized MAX phase with a layered structure after grinding. (i) EDX spectrum of a pellet, revealing the chemical composition of the Ti_3_AlC_2_ MAX phase, and (j) XRD patterns of commercial and as-synthesized Ti_3_AlC_2_ MAX phase powder compared with hexagonal closed-packed Ti_3_AlC_2_ (JCPDS 10-074-8806).

A titanium aluminum carbide (Ti_3_AlC_2_) MAX phase was successfully synthesized using a new pelletizing method that we developed in this study (Fig. S1†). TiC, Ti, and Al were chosen as raw materials, and the powders were prepared for the stoichiometric composition of Ti_3_AlC_2_. These powders were mixed by ball-milling and dried under a nitrogen atmosphere to obtain a uniform and calcined raw-material mixture. The mixture of the powders was shaped into a coin-shaped pellet through cold pressing with an organic binder (*i.e.*, polyvinyl alcohol, PVA), and the inter-particle distance was kept sufficiently short to produce a solid-state reaction. The pellets were then heated at different temperatures ranging from 1400 to 1550 °C in an Ar atmosphere. In previous studies, a homogeneous Ti_3_AlC_1.9_ phase was observed at ≈1300 °C and polycrystalline bulk samples of Ti_3_Al_1.1_C_1.8_ were synthesized reactively by a hot iso-static press at ≈1400 °C.^21,22^ Based on the results, a temperature range over 1400 °C was selected as a synthesis condition for there to be sufficient energy to form the target phase of Ti_3_AlC_2_ with accurate stoichiometry. X-ray diffraction (XRD) analysis confirmed the uniform formation of Ti_3_AlC_2_ at the ambient pressure, as shown in Fig. 1(a). The XRD pattern of the as-milled pellet that was sintered at ≈1400 °C showed peaks for Ti_3_AlC_2_ with TiC (with ≈21.5 vol%) as an impurity. By observing the main peaks and their integral intensities, we measured the volume fraction of Ti_3_AlC_2_ and TiC (Fig. S2(a)–(d)†). As the sintering temperature increased to ≈1450 °C, the Ti_3_AlC_2_ phase increased to 86.6 vol% with TiC being reduced to 13.4 vol%, which indicates that a greater amount of Ti–Al reacted with TiC to produce an interleaved Al layer forming Ti_3_AlC_2_. Based on a previous report that analyzed the reaction path for the formation of Ti_3_AlC_2_ from elemental powders, intermediate phases of non-stoichiometric Ti–Al were formed after Al melting at ≈660 °C.^32,33^ Subsequently, a liquid environment created by the intermediate phases that began to melt at ≈1350 °C favored the reaction with TiC at higher temperature (≈1420 °C) to form Ti_3_AlC_2_. Therefore, it is reasonable to attribute the formation of dense Ti_3_AlC_2_ by the solid–liquid reactions to the chosen sintering condition of the high temperature above the melting point. However, as the sintering temperature further increased to ≈1500 °C, the fraction of Ti_3_AlC_2_ phase slightly decreased to 84.2 vol%. At ≈1550 °C, TiC further increased and a peak for C appeared, showing the same tendency for the reported Ti_3_AlC_2_ phase that decomposes at ≈1580 °C.^32^ Increasing the sintering time from 2 to 4 h did not cause significant changes in terms of the peak position or full width at half maximum (FWHM) except for a relative increase in the intensity of Ti_3_AlC_2_ due to the longer reaction time (Fig. 1(b) and S2(e)–(h)†). PVA was not detected in any of the spectra, suggesting that the organic binder was completely removed above its boiling point (≈228 °C).

The atomic ratio of Ti, Al, and C in the sintered pellet was calculated through energy dispersive X-ray (EDX) analysis (Fig. 1(c)). The Al content was kept fixed, and the stoichiometry ranges explored for Ti_*x*_AlC_*y*_ were *x* = 2.80–3.04 and *y* = 1.75–2.38, suggesting that the sintering conditions, such as temperature, affected the final stoichiometry. Based on these results, an ideal Ti_3_AlC_2_ MAX phase pellet was synthesized using the optimized process conditions. Following sintering at ≈1480 °C for 2 h, the overall color of the pellet changed from dark black to bright grey (Fig. 1(d) and (e)). The layered structure of the MAX phase was observed in scanning electron microscopy (SEM) images of the pellet surface and as-milled powder, revealing that a MAX phase formed within the entire pellet with more than 0.5 g of the raw material (Fig. 1(f)–(h)). X-ray photoelectron spectroscopy (XPS) analysis showed crystalline sheets with a strong covalent bonding of Ti and C and interleaved Al layers that involved relatively weak metallic bonding, as reported in other studies (Fig. S3†).^1–5^ For Ti_3_AlC_2_, Ti 2p peaks centered at ≈455 eV and ≈459 eV could be assigned to the Ti–C bond (formation of Ti-carbide) and Ti(iv) oxide (TiO_2_), respectively.^34^ C 1s peaks centered at ≈281.9 and ≈285 eV were equivalent to Ti–C and C–C bonds, respectively, and Al species occupying the planar sites were also detected at ≈72.3 eV for the Al 2p peak (with oxide species at ≈74.6 eV).^35,36^ The EDX spectrum revealed the compositions of Ti_3_AlC_2_ and the accurate contents of Ti, Al, and C in the pellet (Fig. 1(i)).

XRD analysis showed the successful synthesis of the hexagonal close-packed structure of Ti_3_AlC_2_, corresponding to JCPDS file card no. 10-074-8806 (Fig. 1(j)). To reveal the effect of pelletizing on reducing the impurity phases and fabricating a dense Ti_3_AlC_2_ phase, a comparison with other MAX phases was conducted, namely, commercially available MAX powder with our synthesized one without pelletizing (Table 1). Commercial MAX powder showed unevenly-structured particles, inaccurate stoichiometry, and intermediate phases such as Ti_2_AlC and AlTi_3_ and a large peak of C in the XRD spectrum, leading to a low volume fraction of Ti_3_AlC_2_ (Fig. S4†). Sintering without pelletizing also produced high impurity phases because of the long inter-particle distance in uncompressed and bulky powder, which made the diffusion of atoms difficult (Fig. S5†). In contrast, when a small amount of the powder (0.6 g) was pelletized and heated, two main phenomena during the sintering process, *i.e.*, densification and decrease in the surface area, occurred among the compacted particles, which were verified by pellet shrinkage. The densification could promote the afore-mentioned Ti_3_AlC_2_ formation reaction, resulting in a fully dense Ti_3_AlC_2_ conforming to a previous report for sintering with a wide range of powdered metals through pelletizing and microwave-assisted sintering.^37^ Therefore, the synthesized MAX powder with pelletizing exhibited much fewer impurity phases except for some unreacted TiC, indicating its higher quality and purity.

**Table 1:** Comparison of Ti_3_AlC_2_ MAX phases synthesized by three different methods in terms of volume fraction, atomic ratio, and exfoliation yield of MXenes

Methods		Volume fraction [vol%]		Atomic ratio			Exfoliation yield^a^
		Ti_3_AlC_2_ (MAX phase)	TiC (impurity phase)	Ti (3)	Al (1)	C (2)	
Commercial MAX		32.0	68.0	3.40	1	2.32	0.56 ± 0.14%
Sintering (this work)	Bulky powder (5.84 g)	28.6	71.4	3.04	1	2.77	2.35 ± 1.86%
	Pellet (0.6 g)	91.2	8.8	3.13	1	2.06	55.5 ± 12.8%

a Exfoliation yields were calculated using the equation of (weight of exfoliated MXene flakes)/(weight of used MAX powders).

**Fig. 2:**
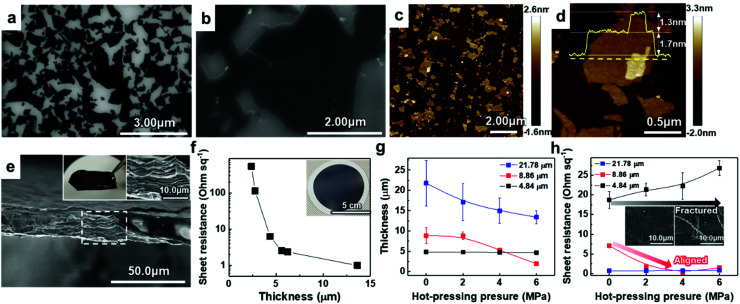
(a and b) SEM images of diluted and dropped Ti_3_C_2_T_*x*_ MXene flakes on a SiO_2_/Si substrate. (c and d) AFM mapping images of the Ti_3_C_2_T_*x*_ MXene flakes. (e) SEM cross-section image of the vacuum-filtrated Ti_3_C_2_T_*x*_ MXene membrane. The insets in (e) are a photograph of a free-standing membrane (left) and HR SEM image of the area marked by a white dashed line (right). (f) Sheet resistance of filtrated Ti_3_C_2_T_*x*_ MXene on PP membranes with different thicknesses. (g) Thickness and (h) sheet resistance of Ti_3_C_2_T_*x*_ MXene membranes hot-pressed with different pressure. Average and standard deviation in (g) were calculated from fifteen measurements within 1 cm of each sample. Sheet resistance in (h) was measured three times per sample and the average and standard deviation were calculated.

To verify the quality of our synthesized MAX precursor, 2D MXene flakes, which have abundant surface functional groups and a large surface area, were produced using a top-down exfoliation method described elsewhere (Fig. S6(a)†).^38^ We used hydrofluoric (HF) acid to selectively etch the Al layers from Ti_3_AlC_2_ without any impurities such as salt by-products. The morphology of the multilayered Ti_3_C_2_T_*x*_ showed that strong interlayer interactions appeared after etching in an HF aqueous solution at room temperature for 24 h followed by centrifugation (Fig. S6(b) and (c)†). Tetramethylammonium hydroxide (TMAOH) was used as an organic intercalant to widen the space and weaken the bonds between the multilayers. Then, sonication followed by centrifugation dispersed the delaminated MXene flakes with dark supernatants present. The MXene flakes, with a strong negative charge on their surface functional groups, were dispersed very stably in DI water, which had remained for several weeks.^39,40^ Non-etched Ti_3_AlC_2_ and Ti_3_C_2_T_*x*_ were separated through centrifugation. When the colloidal solution was investigated by dilution and dropping on SiO_2_/Si substrates, SEM images showed very pure MXene flakes with various lateral sizes of several hundred nanometers (and up to several micrometers), as shown in Fig. 2(a) and (b). The size of the flakes, which affects the application of MXenes,^41^ could be further controlled by optimizing the exfoliation and sintering processes; however, the effect on the resulting MXene flakes is yet to be studied and out of the scope of this study. The high-resolution (HR) SEM image showed that the surface of the flake was very clean without any significant impurities, confirming the purity of our MXene dispersion. When observed through an atomic force microscopy (AFM) mapping image, the MXene was confirmed to be exfoliated in the form of a flake with a small thickness of ≈1.68 ± 0.22 nm, indicating that the exfoliated regions consist of 1–2 layers of MXene (thickness of an individual MXene flake studied by density functional theory calculation and transmission electron microscopy was ≈0.98 nm (ref. 8 and 42)) (Fig. 2(c)). The image of an individual MXene flake and the AFM height profile, measured along the yellow dashed line, show uniform thickness of flakes with dominantly 1–2 layers in the samples (Fig. 2(d)). In addition, thicker regions (with a thickness of ≈3.04 ± 0.11 nm) are also visible in the AFM images, which likely arise from incomplete exfoliation of MXene in suspension.

Solution-processed MXene flakes could be deposited for further measurements and applications. A free-standing MXene membrane was fabricated by vacuum filtration of a highly-concentrated colloidal solution of up to ≈1.2 mg ml^−1^. It was structurally stable and could be handled without any cracking or disintegration (inset in Fig. 2(e)). Orderly stacked flakes that had interlayer gaps were observed by cross-sectional SEM images (Fig. 2(e)). The EDX spectrum showed the elemental composition of Ti and C with an atomic ratio of 3 : 1.72 and O and F as components of the surface functional groups (Fig. S7(a)†). We further investigated the presence and chemical states of foreign species in the samples through XPS (Fig. S3†). For Ti_3_C_2_T_*x*_, Ti 2p peaks at ≈455.8, ≈459.1, and ≈465.0 eV could be attributed to Ti(ii), Ti–O_2−*x*_–F_*x*_ and Ti(iii) bonds, respectively.^43^ A strong XPS peak at ≈459.1 eV (due to the Ti–O_2−*x*_–F_*x*_ bond) proved the presence of large amounts of –O, –OH, and –F functional groups on the surface of MXene flakes after HF etching. Because of selective dissolution of Ti during the HF etching, a C 1s line-scan spectrum exhibited a dominant peak at ≈284.5 eV (because of the C–C bond) as compared to Ti_3_AlC_2_, which corresponds to the graphitic C–C formation.^44^ In addition to the absence of Al in the EDX spectrum, disappearance of the Al 2p peak demonstrated the etching of Al layers of Ti_3_AlC_2_. XRD analysis of the membrane was performed to confirm the structural changes in the MXene phase and its crystallinity (Fig. S7(b)†). The results showed constant peak positions, and the peaks were broadened because of the decrease in the crystallite size. In contrast to the MAX precursor, the intense (104) peak of Ti_3_AlC_2_ at 2*θ* ≈ 38.8° completely disappeared, suggesting the formation of exfoliated 2D Ti_3_C_2_T_*x*_ flakes after Al etching. Compared with that of the MAX precursor, the (002) peak of the MXene, which was initially at 2*θ* ≈ 9°, shifted to a lower angle (*i.e.*, to a larger *d*-spacing, corresponding to exfoliation of MXene).^45^

To utilize the MXene in a large area, a hybrid MXene/polypropylene (PP) membrane with a wide diameter (*φ* = 72 mm) was fabricated by vacuum filtration of MXene on a flexible PP filter (inset in Fig. 2(f)). The thickness of the membrane that could be controlled by varying the amount of vacuum-filtrated solution enabled the electronic properties of the membrane to be tailored, suggesting the metallic nature of the material. It can be seen from Fig. 2(f) that the increase of membrane thickness over 5 μm leads to a dramatic reduction in the sheet resistance below ≈1–5 Ω sq^−1^. The sheet resistance of the membrane could be affected by the alignment of MXene flakes. This is because the electrical conductivity along the vertical direction, having strong Ti–C bonds, is much lower than that in the planar direction.^46^ From the cross-sectional HR SEM images, we found that the membranes consist of highly aligned 2D flakes when the thickness is less than 5 μm; however, they form a continuous conductive network due to the presence of sufficient disordered and nanostructured flakes in the membranes thicker than 5 μm, implying that the system dimensionality changes from 2D to 3D (Fig. S8†).

To align the flakes in the interior of the membrane and minimize the gap between the flakes, which deteriorates the conductivity, hot-pressing was performed on the vacuum-filtrated MXene membrane with multiple levels of pressure. Samples with different initial thicknesses of ≈4.84, ≈8.86, and ≈21.78 μm were pressed into thin, medium, and thick membranes, respectively. Changes in thickness and sheet resistance were measured. The thickness of medium and thick membranes decreased as the pressure of hot-pressing increased (Fig. 2(g)). Interestingly, in a medium-thick membrane, the sheet resistance decreased and reached the minimum value of ≈1–5 Ω sq^−1^ as the applied pressure increased although the pressed membrane had a smaller thickness than the bare membrane (Fig. 2(h)). We assume that this was due to the improved flake alignment and the formation of conductive paths along the planar direction, as in the case of other 2D materials such as graphene.^47^ The cross-sectional SEM images of pressed membranes also show that the gap disappeared and the flakes were densely aligned, compared to the bare membrane (Fig. S9†). On the other hand, the thick membrane, which already had low sheet resistance, showed no significant change. The thin membrane, with no significant change in thickness, showed an increase in sheet resistance due to a membrane fracture (inset in Fig. 2(h)). Therefore, it was confirmed that the conductivity of the MXene membrane could be increased by improving the alignment of the flakes within a moderate thickness range.

**Fig. 3:**
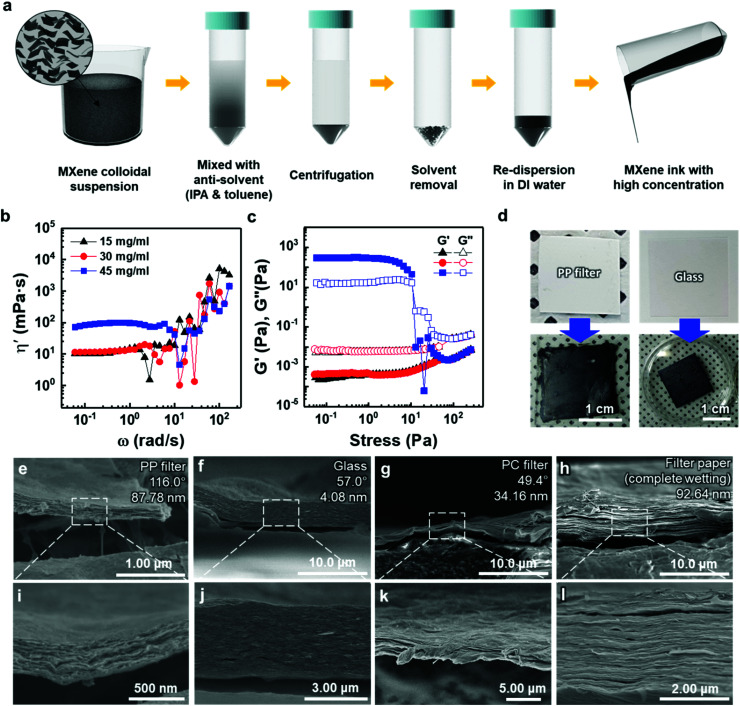
(a) Schematic illustration of the fabrication process of Ti_3_C_2_T_*x*_-MXene ink. (b) Dynamic shear viscosity (*η*′) curves of MXene inks at various concentrations and (c) the storage (*G*′) and loss (*G*′′) moduli curves of Ti_3_C_2_T_*x*_ MXene inks as a function of shear stress at different flake concentrations. (d) Painted Ti_3_C_2_T_*x*_ MXene ink on various substrates using a brush. SEM cross-sectional images of painted Ti_3_C_2_T_*x*_ MXene ink on the (e and i) PP filter, (f and j) glass, (g and k) PC filter, and (h and l) filter paper. Detailed information (*i.e.*, contact angle and root-mean-square surface roughness) on the bare substrates is included on the upper right in (e–h).

To deposit 2D MXene flakes from solution, allowing various devices to be fabricated practically on any surface, MXene flakes should be uniformly deposited with a constant thickness to ensure electrical conductivity and structural stability. Our thickness-dependent results suggest that the vacuum-filtrated membranes are well-stacked and uniform. However, the significant variation in thickness of thicker membranes over ≈5 μm points to the fact that better control of the thickness uniformity of the MXene membranes will be essential if films are to be conformally deposited on various surfaces. Thus, as a new form of MXene applicable to a variety of surfaces, an “MXene ink” that can be easily painted onto various substrates was produced using a method of concentrating purely delaminated flakes, as shown in Fig. 3(a). Precipitating the delaminated MXene flakes was possible using IPA and toluene as anti-solvents with low polarity, as polar solvents disperse MXene more efficiently (Fig. S10(a)†). The sedimented MXene flakes were re-dispersed in DI water by sonication, resulting in MXene ink (Fig. S10(b) and (c)†). The rheological properties of the MXene inks were subsequently investigated to determine the optimal concentration in which inks could flow and be painted over large areas. Although all the inks exhibited Newtonian behavior below the frequency of 1 rad s^−1^, where the dynamic viscosity (*η*′, mPa s) is constant with increasing frequency (*ω*, rad s^−1^) (Fig. 3(b)), it is noteworthy that the *η*′ of the ink with a concentration of 45 mg ml^−1^ was almost one order of magnitude higher than those of the inks prepared at low concentrations. It should also be noted that the dynamic viscosity of all the inks appeared to have increased beyond the frequency of 1 rad s^−1^ with a significant fluctuation. This is because MXene particles in the solution agglomerated at a high shear rate regime, causing phase instability. In addition, the storage (*G*′) and loss (*G*′′) moduli, which are important viscoelastic properties, were investigated by a stress sweep test (Fig. 3(c)). MXene inks, with the concentration of 15 and 30 mg ml^−1^, showed low loss and storage moduli with no fluctuation with increasing stress. While the MXene ink with a concentration of 45 mg ml^−1^ showed an abrupt decrease, which may be due to the fact that MXene flakes became aggregated at high shear stress over a linear viscoelastic (LVE) region (∼10 Pa). To check the viability for painting, the MXene inks (15, 30, and 45 mg ml^−1^ concentrations) were painted on several kinds of substrates with hydrophobic and hydrophilic surfaces. A thick ink with a concentration of 45 mg ml^−1^ could not be painted on entire substrates due to a lack of flow (Fig. S11†). Therefore, for uniform painting on substrates with rough surface morphology or large area, to prevent the aggregation of MXene flakes, liquid-like inks with concentration of less than 45 mg ml^−1^ were considered appropriate due to their stable rheological properties and fluidity; even though a high concentration of MXene ink is advantageous for painting a thick MXene flake layer. In this regard, an MXene ink with a concentration of 30 mg ml^−1^ was adopted for painting applications. The ink was painted well on hydrophobic substrates, such as the PP filter and glass, using a brush (Fig. 3(d)).

The cross-sectional SEM images of painted MXene ink on a PP filter, glass (DURAN, borosilicate glass (D263)), a hydrophilic polycarbonate (PC) filter, and a filter paper (Hyundai micro, HM.00205110) showed that the MXene ink was painted with a uniform thickness, and the flakes were stacked laterally as compared to others made of a whole sediment that possibly contained multilayer MXenes or impurities (Fig. 3(e)–(l)). The lower magnification cross-sectional SEM images of these specimens showed their thickness uniformity over a wide range (Fig. S12†). In particular, on a filter paper, MXene was first coated on a fiber and then stacked up, which indicated that the MXene ink was painted conformally based on the morphology of the underlying substrate (Fig. S13†). It was also confirmed through XRD analysis that the flakes were deposited in a manner similar to that of the vacuum-filtrated membrane (Fig. S7(b)†).

**Fig. 4:**
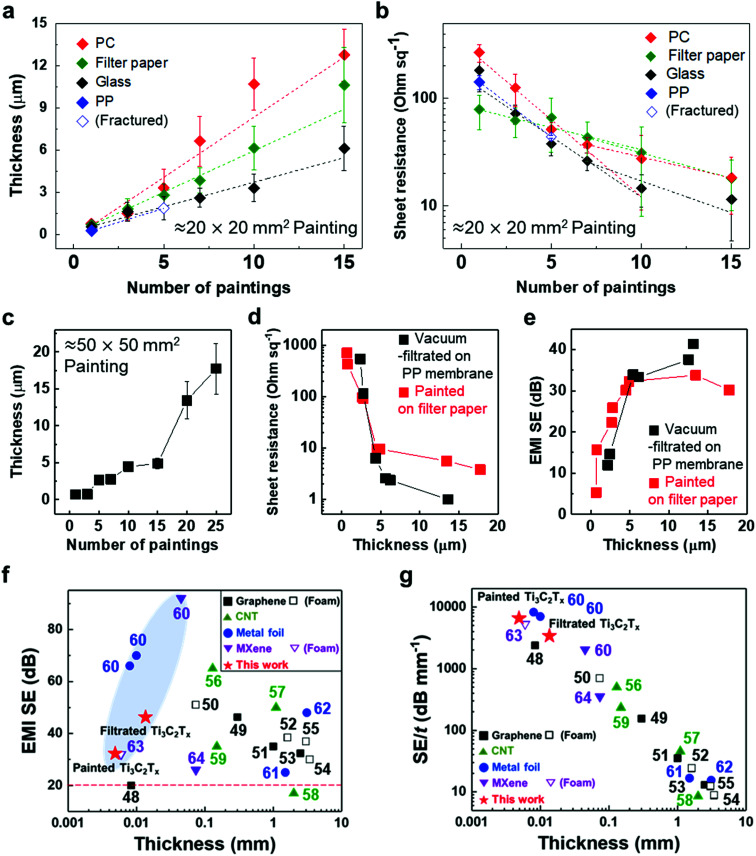
(a) Thickness and (b) sheet resistance of painted Ti_3_C_2_T_*x*_ MXene ink as a function of number of paintings. (c) Thickness of painted Ti_3_C_2_T_*x*_ MXene on filter paper with a different number of paintings. (d) Sheet resistance and (e) EMI SE of painted Ti_3_C_2_T_*x*_ MXene/filter paper with different thicknesses. (f) EMI SE and (g) EMI SE/*t versus* thickness of previously reported different materials. Detailed EMI shielding data are presented in Table S1 in the ESI.† The horizontal red dashed line in (f) indicates basic requirements for commercialization. Average and standard deviation of thickness in (a) and (c) were calculated from 15 measurements within 1 cm of each of the three samples, with the same number of paintings. Sheet resistance in (b) was measured with three samples with the same number of paintings and the average and standard deviation were calculated.

To validate the applicability of MXene ink painting on desired substrates, the thickness and sheet resistance of the painted Ti_3_C_2_T_*x*_ MXene inks on various substrates having areas of ≈20 × 20 mm^2^ were plotted as a function of the number of paintings, as shown in Fig. 4(a) and (b). The results showed that the thickness increased almost linearly with the number of paintings and the sheet resistance exponentially decreased with the thickness of the flake layer. We note that MXene ink was painted more densely on glass having small roughness (≈4.08 nm), leading to the fabrication of the MXene membrane with a high electrical conductivity (with a thickness of ≈7.7 μm and a sheet resistance of ≈4.98 Ω sq^−1^). It is worthwhile to note that a larger deviation in the thickness and sheet resistance of the painted Ti_3_C_2_T_*x*_ MXene inks on the PC filter, which was thin (with a thickness of ≈4.7 μm) and easily crumpled, was observed compared to those on other substrates. In addition, the painted Ti_3_C_2_T_*x*_ MXene inks on the PP filter (with small pore size of ≈0.2 μm and hydrophobic surface) could not be deposited more than 5 times because of the aggregation and spills of the flakes, ending up with a fracture in the film (Fig. S14†).

**Fig. 5:**
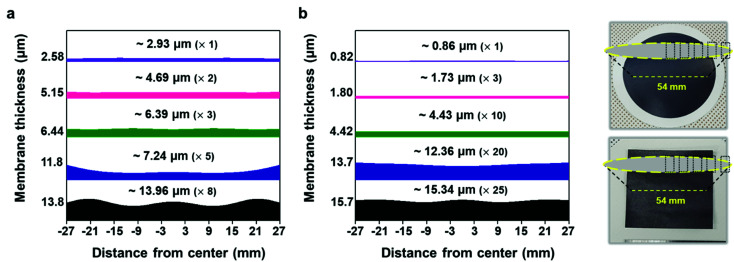
Graph showing the cross-section thicknesses of the (a) vacuum-filtrated MXene membrane and (b) painted MXene ink. It shows the thickness non-uniformity of the MXene membrane deposited by two different ways over a large area of ≈54 mm in diameter. The numbers above the spectrum indicate the average thicknesses (the number of vacuum filtration or painting processes) of the MXene membranes. Photographs show the corresponding areas where the thicknesses were measured in the vacuum-filtrated MXene membrane (top) and painted MXene ink (bottom).

With the structure of the deposited MXene flakes being similar to the vacuum-filtrated membrane, our painted MXene ink with much better thickness uniformity is expected to be used in many potential applications, as shown in Fig. 5. For comparison with the EMI shielding performance of the hybrid MXene/PP membrane, MXene ink was painted on a filter paper over a large area of ≈40 × 40 mm^2^. We note that the different substrates were introduced to avoid the aggregation and penetration of MXenes and the substrates did not affect the EMI shielding effectiveness (SE) due to their insulating properties. Even in the large area, the thickness of the MXene could be increased uniformly by ink painting except at very high values over 15 (Fig. 4(c)). The thickness played a crucial role in the EMI SE of the membrane as the effective area through which the wave passes. For electrically conductive materials, EMI SE can be theoretically represented by the conventional Simon formalism;1
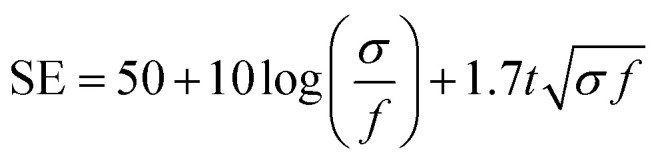
where *σ* [S cm^−1^] is the electrical conductivity, *f* [MHz] is the frequency and *t* [cm] is the thickness of shielding materials. As expected, the EMI shielding performance of the MXene membrane exhibited a strong dependence on the metallic MXene layer thickness. The electrical conductivity of the membrane, which is another important property that reflects the EMI waves, was also plotted, and the thicker MXene showed much lower sheet resistance in both vacuum filtration and painting (Fig. 4(d)). Consequently, the painted MXene on filter paper showed a similar tendency for EMI SE with the vacuum-filtrated MXene (Fig. 4(e)). Comparison of EMI SE *versus* thickness with previously reported different materials showed that our filtrated/painted MXene had excellent performance exceeding the basic requirements for commercialization (Fig. 4(f)).^48–64^ When the EMI SE in the X-band was evaluated over a large area of ≈40 × 40 mm^2^, the pure MXene membrane on the PP substrate showed shielding due to absorption (≈58.5%) and reflection (≈41.5%) and a high total SE of up to ≈46.3 dB with the MXene thickness of ≈13.6 μm (Fig. S15†). In addition, a more realistic parameter for determining the effectiveness of a material in membrane and coating applications is to divide EMI SE by the material thickness (SE/*t*). We note that EMI SE/*t* values for our filtrated and painted MXene films are much higher than those for other coating materials of different categories (Fig. 4(g)). For example, a painted MXene sample without any additives gives a SE/*t* of ≈6591.8 dB mm^−1^, which is at least several times higher than those of the other materials reported so far. Moreover, we confirmed that our synthesized MXene flakes were of an excellent quality as compared to the previously reported ones on specific SE/*t* (SSE/*t*) values such as graphene, CNT, metal foils, or MXenes (Table S1†).

## 3. Conclusions

In this paper, we demonstrated a facile approach to synthesize a highly purified precursor MAX phase and controllable deposition of 2D MXene flakes exfoliated from the precursor using vacuum filtration and a further alignment process. Moreover, the development of a direct painting process with an ink composed only of delaminated-MXene flakes enabled the 2D flakes to be easily deposited on various substrates with improved thickness uniformity and laterally stacked morphology. High conductivity and improved EMI shielding ability of painted MXene films without any additives were achieved, which are more valued in producing industrial-scale MXene membranes and coatings that are applicable to electronic and energy storage devices. Notably, our synthesized MXene flakes were of excellent quality as compared to previously reported ones on EMI shielding effectiveness using other synthetic materials. This work not only proposes a simple method to create high-quality precursors for MXenes with desired chemical compositions, but also expands the availability of the MXene ink by providing a new manufacturing method with rheological characterization. Our results could provide a route for the translation of MXene-based research from the fundamental to the technological realm.

## 4. Materials and methods

### 4.1 Materials

Tetramethylammonium hydroxide solution (TMAOH; ACS reagent; 1.0 ± 0.02 M in H_2_O) was purchased from Sigma-Aldrich. Hydrofluoric acid (HF; ACS reagent; 48–51% solution in H_2_O) was purchased from Acros Organics. Further, Ti (44 μm average particle size, 99.5% purity) and TiC (2 μm average particle size, 99.5% purity) were purchased from Alfa Aesar. Al (30 μm average particle size, 99.7% purity) was purchased from US Research Nanomaterials, Inc. All elements and chemicals were used without any further purification.

### 4.2 Synthesis of the parent Ti_3_AlC_2_ MAX phase

TiC, Ti, and Al powders were mixed by ball milling for 18 h using ethanol and zirconia balls in a high-density polyethylene (HDPE) bottle at a molar ratio of 2 : 1 : 1; subsequently, these were used as raw materials for the Ti_3_AlC_2_ MAX phase. Ethanol was dried under a nitrogen atmosphere for 24 h at 100 °C. The calcined mixture was milled using an agate pestle and mortar and pelletized with PVA as an organic binder using cold-pressing under 4 MPa of pressure. The pellet was heated in a tungsten crucible at 5 °C min^−1^ to 1480 °C and held for 2 h under a flow of argon. After cooling, the sintered pellet was milled for further measurements and exfoliation.

### 4.3 Synthesis of Ti_3_C_2_T_*x*_ MXene

The 2D Ti_3_C_2_T_*x*_ flakes were delaminated using a previously reported top-down exfoliation method.^38^ Specifically, 0.5 g of synthesized Ti_3_AlC_2_ MAX powder was added into 20 ml of 25% HF aqueous solution at 25 °C under constant stirring for 24 h to selectively etch the Al. The obtained suspension was washed with deionized water several times and centrifuged to obtain a multilayered Ti_3_C_2_T_*x*_ sediment. Subsequently, 5 ml of TMAOH with 50 ml of deionized water were added and stirred for 48 h at 25 °C. The mixture was washed twice *via* centrifugation and the sediment of intercalated Ti_3_C_2_T_*x*_ was sonicated for 45 min. After centrifugation for 1 h at 3500 rpm, the upper layer of the solution (35 ml of 50 ml centrifuge tube) was collected as a colloidal solution that comprised only delaminated MXene flakes.

### 4.4 Fabrication of the vacuum-filtrated MXene membrane

10 ml of a colloidal solution of MXene flakes with a concentration of 1.5 mg ml^−1^ was vacuum-filtrated on the PP membrane filter (pore size of 0.2 μm, Sterlitech) and dried under a nitrogen atmosphere for 24 h at 70 °C. The thickness of the MXene was controlled by the amount of vacuum-filtrated solution added. Alignment of MXene flakes was achieved using additional hot-pressing at 2, 4, and 6 MPa, at 80 °C.

### 4.5 Fabrication of Ti_3_C_2_T_*x*_ MXene ink

20 ml of a colloidal solution of MXene flakes with a concentration of 1.5 mg ml^−1^ was mixed with 100 ml of anti-solvents (volume fraction of IPA : toluene = 2.25 : 1) and precipitated by centrifugation for 10 min at 7830 rpm. The mixed solvents were dried under vacuum for 30 min using a rotary pump and the sedimented MXene flakes were re-dispersed in 1 ml of deionized water using sonication to obtain MXene ink with a high concentration of 30 mg ml^−1^. The inks at concentrations of 15 and 45 mg ml^−1^ were manufactured by the same method, except for the initial amount of solution. The MXene ink was painted with a brush onto various substrates heated at ∼60 °C using a hot plate for rapid solvent evaporation.

### 4.6 Characterization

The surface morphologies and EDX spectra of synthesized MAX powder and exfoliated MXene flakes were obtained by cold FE-SEM (Hitachi High-Technologies S-4800). The thicknesses of the vacuum-filtrated MXene membrane and painted MXene ink were measured *via* the cross-sectional SEM images using ImageJ software. XRD patterns were recorded on a high-power X-ray diffractometer (Rigaku D/MAX2500 V, Cu Kα radiation, *λ* = 1.54178 Å). AFM mapping images of delaminated MXene flakes were obtained using an AFM (Veeco MultiMode V) in tapping mode. XPS measurements were conducted on an X-ray photoelectron spectrometer (ThermoFisher K-alpha). Electrical conductivity measurements were performed at room temperature using a standard four-probe method on a Keithley 2400 sourcemeter measurement system. All samples were cut into a square shape with an area of ∼20 × 20 mm^2^ and a silver paste was coated at the edges for ease of measurement. A four-pin probe was tightly contacted with the silver paste on vacuum-filtrated MXene or painted MXene ink. Subsequently, the sheet resistance was measured.

The dynamic rheological properties of the Ti_3_C_2_T_*x*_ MXene inks with concentrations of 15, 30, and 45 mg ml^−1^ were measured using a rotational rheometer (Haake MARS III, Thermo Scientific) at 25 °C; this device had coaxial cylinder geometry with the CC16 DIN Ti accessory. To prevent evaporation of the solvent during measurement, the upper surface of the samples in the cylinder was covered by a mineral oil. The frequency sweep tests were carried out at a constant stress of 0.5 Pa and the stress sweep tests were conducted over the range of 0.05–300 Pa at a rate of 0.5 rad s^−1^.

The EMI shielding properties were measured using a vector network analyzer (Keysight N5222B) using the waveguide method within the range of 8.2–12.4 GHz. All the samples were cut into a square shape with a size of ≈41 × 41 mm^2^ for measurements. The total electromagnetic interference shielding effectiveness (SE_T_) of the vacuum-filtrated Ti_3_C_2_T_*x*_ MXene/PP membrane filter and painted Ti_3_C_2_T_*x*_/filter paper refers to the logarithm of the ratio of incident power (*P*_i_) to transmitted power (*P*_t_) of radiation, which was the sum of the absorption (SE_A_), reflection (SE_R_) and multiple reflections (SE_M_):^60^2
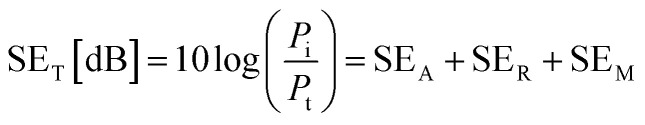


The corresponding SE_A_ and SE_R_ could be calculated as:3
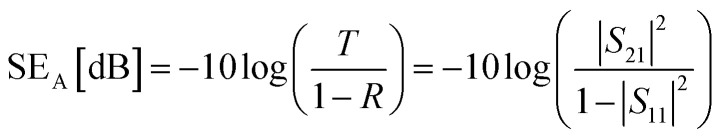

4 SE_R_ [dB] = −10 log(1 − *R*) = −10 log(1 − |*S*_11_|^2^)

where the power coefficients of reflection (*R*), transmission (*T*), and absorption (*A*) are calculated using the scattering parameters (*S*_11_ and *S*_21_).

## Conflicts of interest

The authors declare no conflict of interest.

## Supplementary Material

NA-003-D0NA00398K-s001

## References

[cit1] Anasori B., Lukatskaya M. R., Gogotsi Y. (2017). Nat. Rev. Mater..

[cit2] Hong Ng V. M., Huang H., Zhou K., Lee P. S., Que W., Xu J. Z., Kong L. B. (2017). J. Mater. Chem. A.

[cit3] Xiong D., Li X., Bai Z., Lu S. (2018). Small.

[cit4] Naguib M., Kurtoglu M., Presser V., Lu J., Niu J., Heon M., Hultman L., Gogotsi Y., Barsoum M. W. (2011). Adv. Mater..

[cit5] Mashtalir O., Naguib M., Mochalin V. N., Dall'Agnese Y., Heon M., Barsoum M. W., Gogotsi Y. (2013). Nat. Commun..

[cit6] Dillon A. D., Ghidiu M. J., Krick A. L., Griggs J., May S. J., Gogotsi Y., Barsoum M. W., Fafarman A. T. (2016). Adv. Funct. Mater..

[cit7] Lukatskaya M. R., Mashtalir O., Ren C. E., Dall'Agnese Y., Rozier P., Taberna P. L., Naguib M., Simon P., Barsoum M. W., Gogotsi Y. (2013). Science.

[cit8] Ghidiu M., Lukatskaya M. R., Zhao M.-Q., Gogotsi Y., Barsoum M. W. (2015). Nature.

[cit9] Li J., Yuan X., Lin C., Yang Y., Xu L., Du X., Xie J., Lin J., Sun J. (2017). Adv. Energy Mater..

[cit10] Zhou Z., Panatdasirisuk W., Mathis T. S., Anasori B., Lu C., Zhang X., Liao Z., Gogotsi Y., Yang S. (2018). Nanoscale.

[cit11] Zhang J., Zhao Y., Guo X., Chen C., Dong C.-L., Liu R.-S., Han C.-P., Li Y., Gogotsi Y., Wang G. (2018). Nat. Catal..

[cit12] Kim S. J., Koh H.-J., Ren C. E., Kwon O., Maleski K., Cho S.-Y., Anasori B., Kim C.-K., Choi Y.-K., Kim J., Gogotsi Y., Jung H.-T. (2018). ACS Nano.

[cit13] Hantanasirisakul K., Gogotsi Y. (2018). Adv. Mater..

[cit14] Xu C., Wang L., Liu Z., Chen L., Guo J., Kang N., Ma X.-L., Cheng H.-M., Ren W. (2015). Nat. Mater..

[cit15] Zhou J., Zha X., Chen F. Y., Ye Q., Eklund P., Du S., Huang Q. (2016). Angew. Chem., Int. Ed..

[cit16] VahidMohammadi A., Mojtabavi M., Caffrey N. M., Wanunu M., Beidaghi M. (2019). Adv. Mater..

[cit17] Kim S.-Y., Kwak J., Ciobanu C. V., Kwon S.-Y. (2019). Adv. Mater..

[cit18] Feng A., Yu Y., Wang Y., Jiang F., Yu Y., Mi L., Song L. (2017). Mater. Des..

[cit19] Li Z., Wang L., Sun D., Zhang Y., Liu B., Hu Q., Zhou A. (2015). Mater. Sci. Eng., B.

[cit20] Feng A., Yu Y., Jiang F., Wang Y., Mi L., Yu Y., Song L. (2017). Ceram. Int..

[cit21] Tzenov N. V., Barsoum M. W. (2004). J. Am. Ceram. Soc..

[cit22] Zhou A. G., Barsoum M. W. (2010). J. Alloys Compd..

[cit23] Scheibe B., Kupka V., Peplińska B., Jarek M., Tadyszak K. (2019). Materials.

[cit24] Shuck C. E., Han M., Maleski K., Hantanasirisakul K., Kim S. J., Choi J., Reil W. E. B., Gogotsi Y. (2019). ACS Appl. Nano Mater..

[cit25] Zhou A., Wang C. A., Hunag Y. (2003). J. Mater. Sci..

[cit26] Yeh C. L., Kuo C. W., Chu Y. C. (2010). J. Alloys Compd..

[cit27] Melchior S. A., Raju K., Ike I. S., Erasmus R. M., Kabongo G., Sigalas I., Iyuke S. E., Ozoemena K. I. (2018). J. Electrochem. Soc..

[cit28] Tan Y., Chen C., Li S., Han X., Xue J., Liu T., Zhou X., Peng S., Zhang H. (2019). Ceram. Int..

[cit29] Quain E., Mathis T. S., Kurra N., Maleski K., Van Aken K. L., Alhabeb M., Alshareef H. N., Gogotsi Y. (2018). Adv. Mater. Technol..

[cit30] Zhang C., Park S.-H., Seral-Ascaso A., Barwich S., McEvoy N., Boland C. S., Coleman J. N., Gogotsi Y., Nicolosi V. (2019). Nat. Commun..

[cit31] Yang W., Yang J., Byun J. J., Moissinac F. P., Xu J., Haigh S. J., Domingos M., Bissett M. A., Dryfe R. A. W., Barg S. (2019). Adv. Mater..

[cit32] Pietzka M. A., Schuster J. C. (1994). J. Phase Equilib..

[cit33] Wang X., Zhou Y. (2002). J. Mater. Chem..

[cit34] Halim J., Cook K. M., Naguib M., Eklund P., Gogotsi Y., Rosen J., Barsoum M. W. (2016). Appl. Surf. Sci..

[cit35] Kisi E. H., Crossley J. A. A., Myhra S., Barsoum M. W. (1998). J. Phys. Chem. Solids.

[cit36] Myhra S., Crossley J. A. A., Barsoum M. W. (2001). J. Phys. Chem. Solids.

[cit37] Roy R., Agrawal D., Cheng J., Gedevanlshvili S. (1999). Nature.

[cit38] Alhabeb M., Maleski K., Anasori B., Lelyukh P., Clark L., Sin S., Gogotsi Y. (2017). Chem. Mater..

[cit39] Ma Z., Zhou X., Deng W., Lei D., Liu Z. (2018). ACS Appl. Mater. Interfaces.

[cit40] Hu M., Hu T., Li Z., Yang Y., Cheng R., Yang J., Cui C., Wang X. (2018). ACS Nano.

[cit41] Maleski K., Ren C. E., Zhao M.-Q., Anasori B., Gogotsi Y. (2018). ACS Appl. Mater. Interfaces.

[cit42] Wang X., Shen X., Gao Y., Wang Z., Yu R., Chen L. (2015). J. Am. Chem. Soc..

[cit43] Yan H., Li W., Li H., Fan X., Zhu M. (2019). Prog. Org. Coat..

[cit44] Lukatskaya M. R., Halim J., Dyatkin B., Naguib M., Buranova Y. S., Barsoum M. W., Gogotsi Y. (2014). Angew. Chem., Int. Ed..

[cit45] Lipatov A., Alhabeb M., Lukatskaya M. R., Boson A., Gogotsi Y., Sinitskii A. (2016). Adv. Electron. Mater..

[cit46] Zhou Y. C., Wang X. H., Sun Z. M., Chen S. Q. (2001). J. Mater. Chem..

[cit47] Yousefi N., Sun X., Lin X., Shen X., Jia J., Zhang B., Tang B., Chan M., Kim J.-K. (2014). Adv. Mater..

[cit48] Shen B., Zhai W., Zheng W. (2014). Adv. Funct. Mater..

[cit49] Song W.-L., Fan L.-Z., Cao M.-S., Lu M.-M., Wang C.-Y., Wang J., Chen T.-T., Li Y., Hou Z.-L., Liu J., Sun Y.-P. (2014). J. Mater. Chem. C.

[cit50] Li Y., Shen B., Pei X., Zhang Y., Yi D., Zhai W., Zhang L., Wei X., Zheng W. (2016). Carbon.

[cit51] Yan D.-X., Pang H., Xu L., Bao Y., Ren P.-G., Lei J., Li Z.-M. (2014). Nanotechnology.

[cit52] Song Q., Ye F., Yin X., Li W., Li H., Liu Y., Li K., Xie K., Li X., Fu Q., Cheng L., Zhang L., Wei B. (2017). Adv. Mater..

[cit53] Yan D.-X., Pang H., Li B., Vajtai R., Xu L., Ren P.-G., Wang J.-H., Li Z.-M. (2015). Adv. Funct.
Mater..

[cit54] Chen Z., Xu C., Ma C., Ren W., Cheng H.-M. (2013). Adv. Mater..

[cit55] Song W.-L., Guan X.-T., Fan L.-Z., Cao W.-Q., Wang C.-Y., Cao M.-S. (2015). Carbon.

[cit56] Lu S., Shao J., Ma K., Chen D., Wang X., Zhang L., Meng Q., Ma J. (2018). Carbon.

[cit57] Al-Saleh M. H., Saadeh W. H., Sundararaj U. (2013). Carbon.

[cit58] Liu Z., Bai G., Huang Y., Ma Y., Du F., Li F., Guo T., Chen Y. (2007). Carbon.

[cit59] Wang L.-L., Tay B.-K., See K.-Y., Sun Z., Tan L.-K., Lua D. (2009). Carbon.

[cit60] Shahzad F., Alhabeb M., Hatter C. B., Anasori B., Hong S. M., Koo C. M., Gogotsi Y. (2016). Science.

[cit61] Ji K., Zhao H., Zhang J., Chen J., Dai Z. (2014). Appl. Surf. Sci..

[cit62] Ameli A., Nofar M., Wang S., Park C. B. (2014). ACS Appl. Mater. Interfaces.

[cit63] Liu J., Zhang H.-B., Sun R., Liu Y., Liu Z., Zhou A., Yu Z.-Z. (2017). Adv. Mater..

[cit64] Cao W.-T., Chen F.-F., Zhu Y.-J., Zhang Y.-G., Jiang Y.-Y., Ma M.-G., Chen F. (2018). ACS Nano.

