# Supplementary material for: Synthesis of high quality 2D carbide MXene flakes using a highly purified MAX precursor for ink applications
Source: Nanoscale Adv. 2020 Nov 23;3(2):517–27. doi: 10.1039/d0na00398k (PMC9417611; doi:10.1039/d0na00398k)
Supplement: NA-003-D0NA00398K-s001 [file NA-003-D0NA00398K-s001.pdf]

Electronic Supplementary Material (ESI) for Nanoscale Advances.
This journal is © The Royal Society of Chemistry 2020

Supporting Information for

Synthesis of High Quality 2D Carbide MXene Flakes Using Highly-Purified MAX Precursors for Ink Applications

Shi-Hyun Seok[1], Seungjun Choo[1], Jinsung Kwak[1], Hyejin Ju[1], Ju-Hyoung Han[1], Woo-Seok Kang[1], Joonsik Lee[2], Se-Yang Kim[1], Do Hee Lee[1], Jungsoo Lee[1], Jaewon Wang[1], Seunguk Song[1], Wook Jo[1], Byung Mun Jung[2], Han Gi Chae[1]*, Jae Sung Son[1]*, Soon-Yong Kwon[1]*

[1]School of Materials Science and Engineering & Center for Future Semiconductor Technology (FUST), Ulsan National Institute of Science and Technology (UNIST), Ulsan, 44919, Korea

[2]Composites Research Division, Korea Institute of Materials Science (KIMS), Changwon, 51508, Korea

*E-mails: hgchae@unist.ac.kr, jsson@unist.ac.kr, sykwon@unist.ac.kr

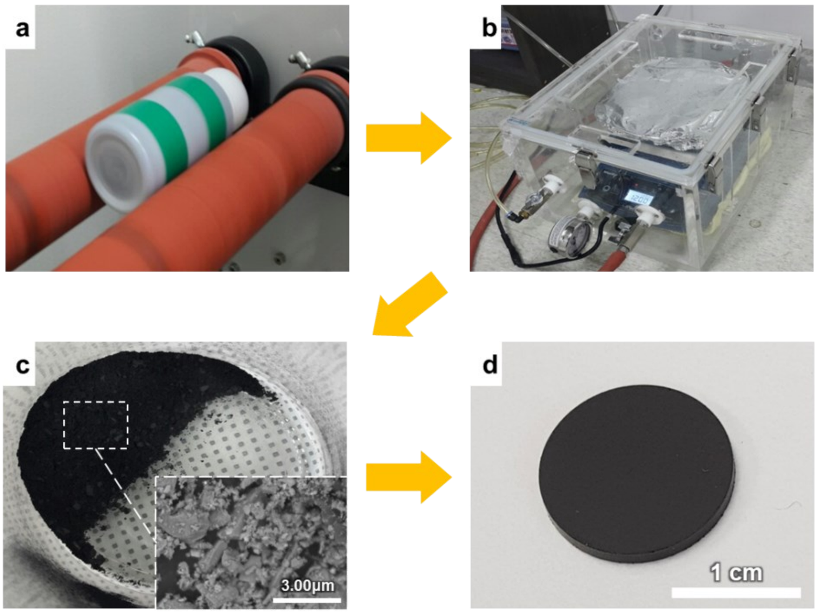

Fig. S1. Preparation of cold-pressed pellets. (a) Ball-milling of TiC, Ti, and Al mixed powders using zirconia balls in plastic jars in a rolling machine, (b) drying in an N_2 chamber, (c) grinding raw powders (inset: SEM image of mixed powders of TiC, Ti, and Al), and (d) a photograph of a cold-pressed pellet.

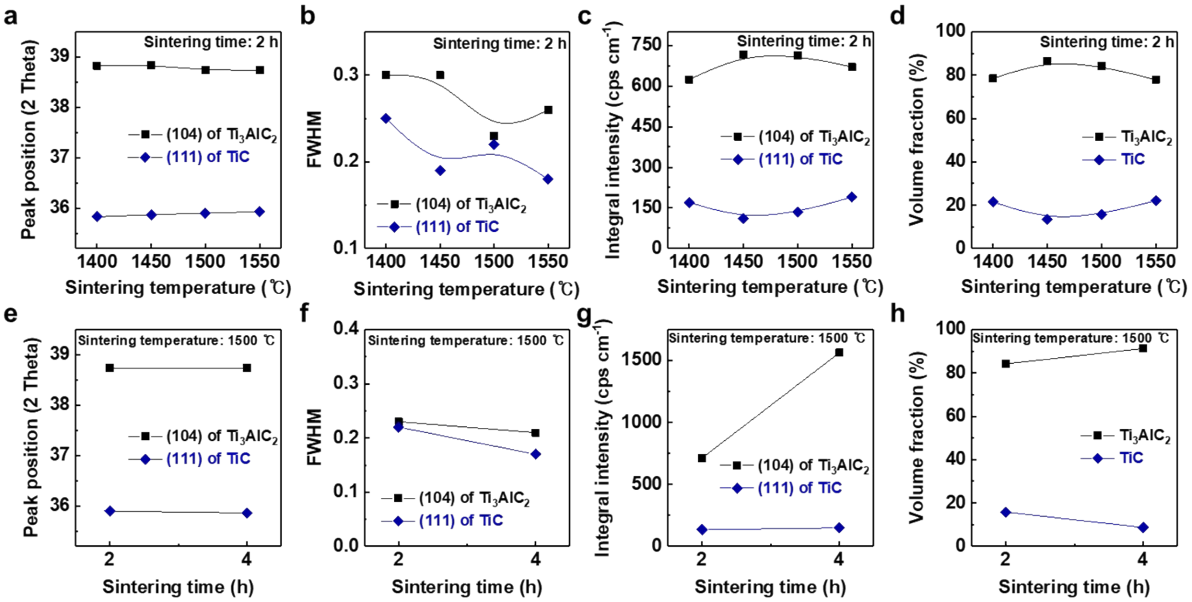

Fig. S2. Phase analysis of as-milled pellets sintered at different temperatures and times: (a, e) main peak position; (b, f) FWHM, (c, g) integral intensity, and (d, h) volume fraction of Ti_3AlC_2 and TiC.

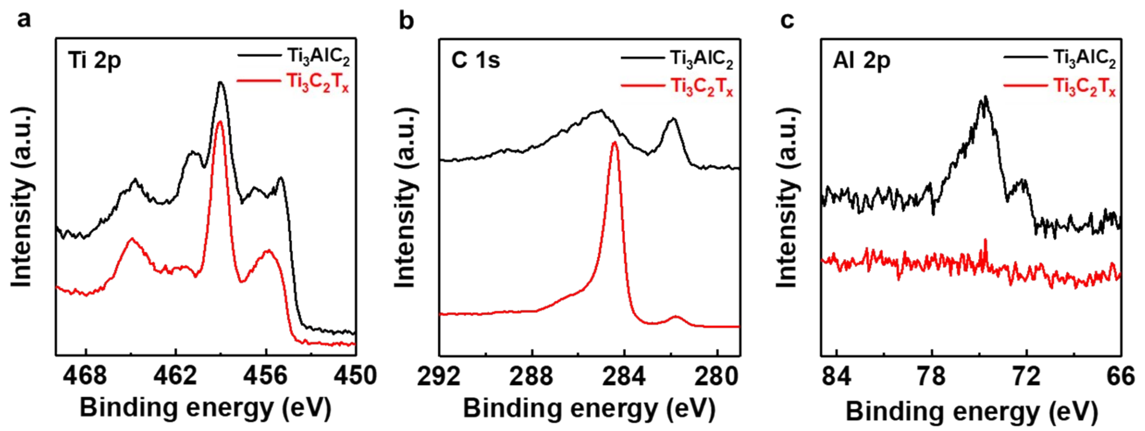

Fig. S3. XPS analysis of as-synthesized Ti_3AlC_2 MAX powder and $Ti_3C_2T_x$ MXene powder: (a) Ti *2p*, (b) C *1s*, and (c) Al *2p* XPS spectrum.

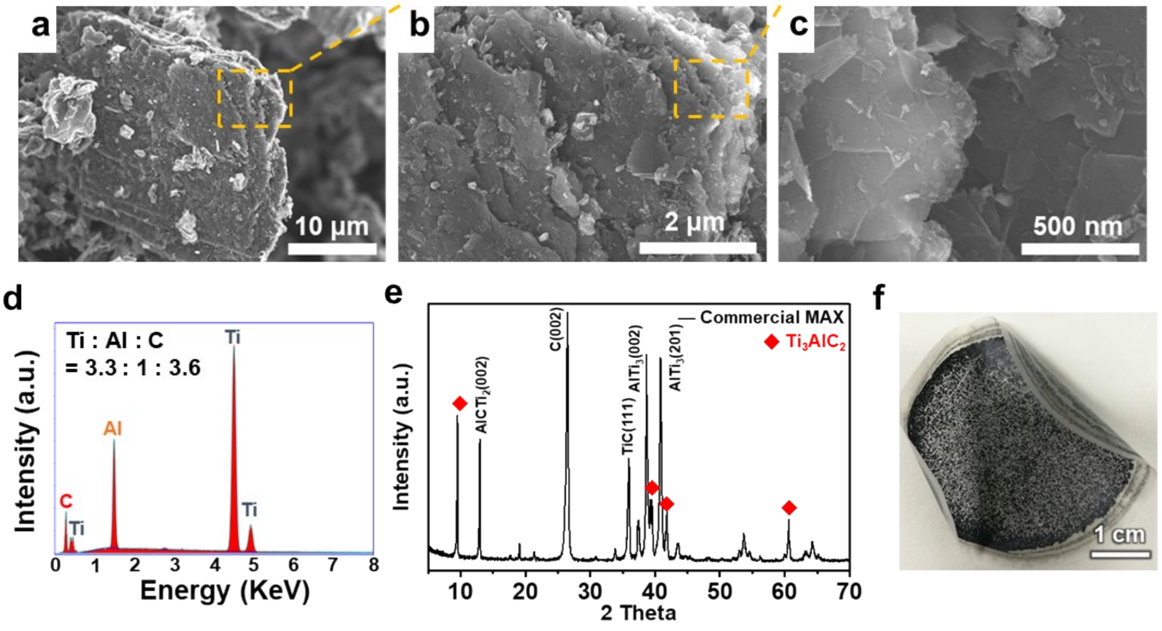

Fig. S4. (a–c) SEM images of commercially available Ti_3AlC_2 MAX powder showing the low fraction of layered structure. (d) EDX spectrum of commercial MAX powder, revealing an inaccurate stoichiometry for Ti_3AlC_2. (e) XRD pattern of commercial MAX powder with many intermediated phases. (f) A photograph of PC filter membrane after vacuum filtration of MXene colloidal solution exfoliated from commercial MAX powder, revealing the low exfoliation yield of MXenes.

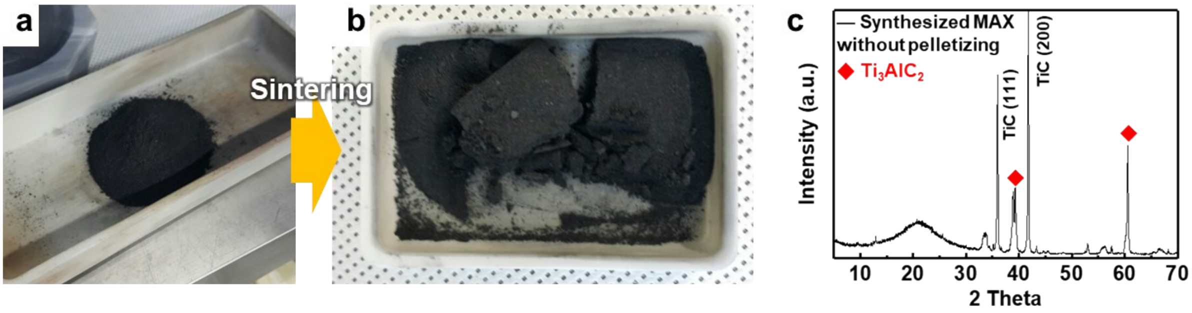

Fig. S5. Photographs of (a) prepared bulky powder and (b) after the sintering process at ≈1,480 °C for 2 h. (c) XRD pattern of as-synthesized Ti_3AlC_2 MAX phase powder without pelletizing having a high amount of TiC as an impurity phase.

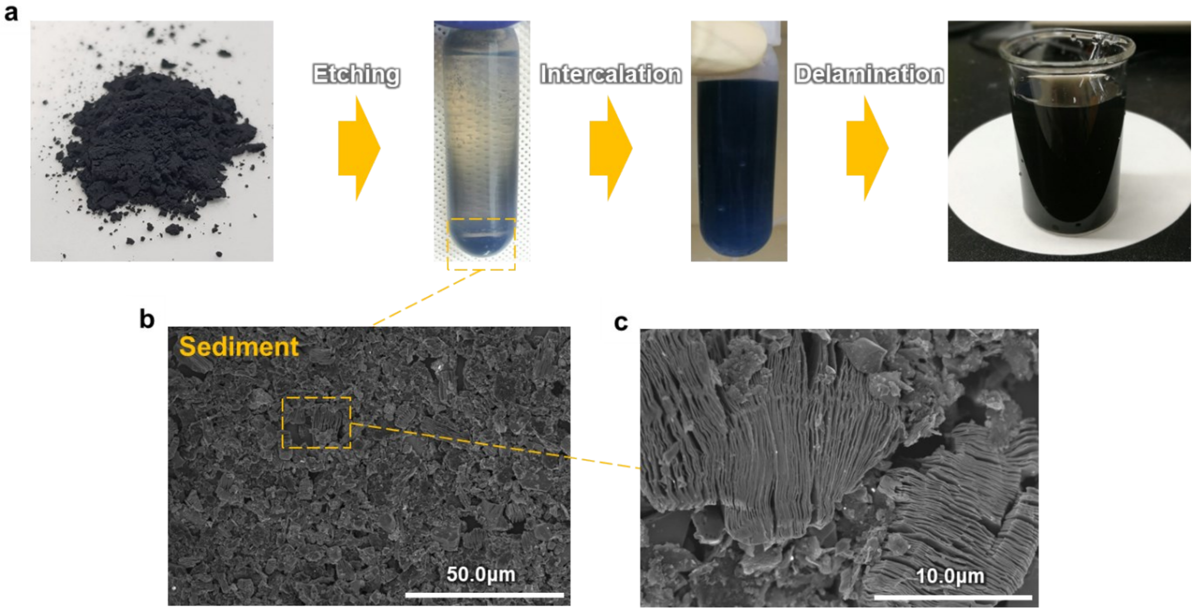

Fig. S6. (a) Exfoliation process for synthesis of 2D $Ti_3C_2T_x$ MXene flakes. (b, c) SEM images of etched powder resulting in a multilayer $Ti_3C_2T_x$.

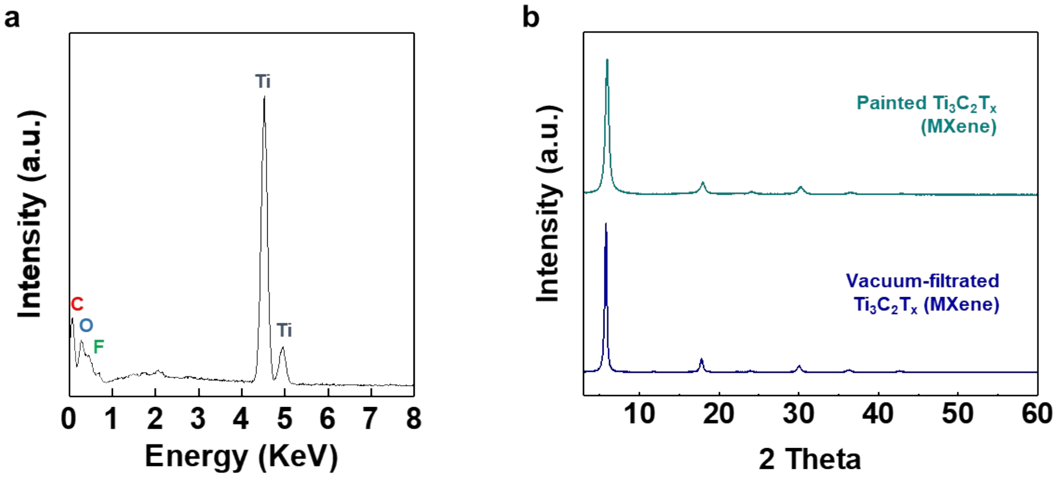

Fig. S7. (a) EDX spectrum of $Ti_3C_2T_x$ MXene. (b) XRD patterns of vacuum-filtrated $Ti_3C_2T_x$ MXene membrane (bottom) and painted $Ti_3C_2T_x$ MXene ink (top).

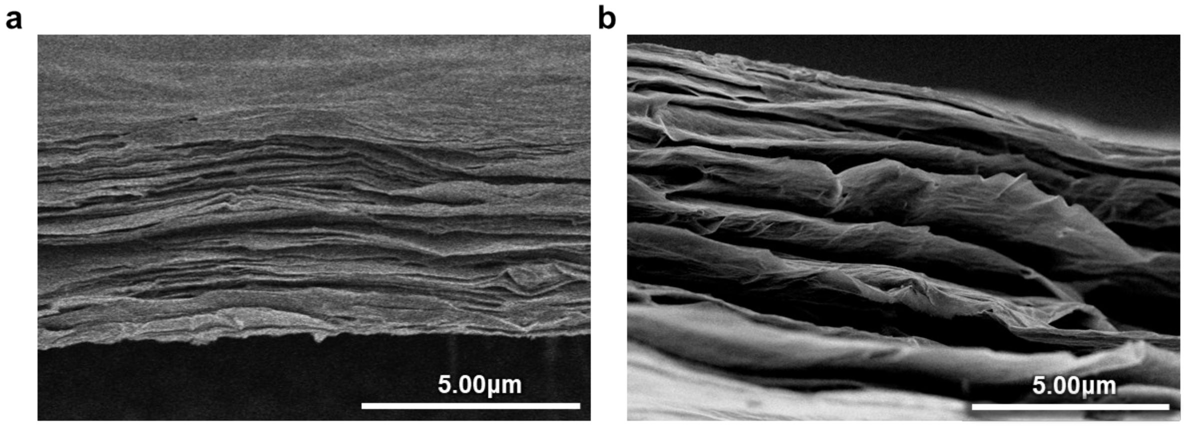

Fig. S8. Cross-sectional SEM images of (a) a thin membrane with an average thickness of ≈4.7 μm and (b) a thick membrane with an average thickness of ≈14.1 μm.

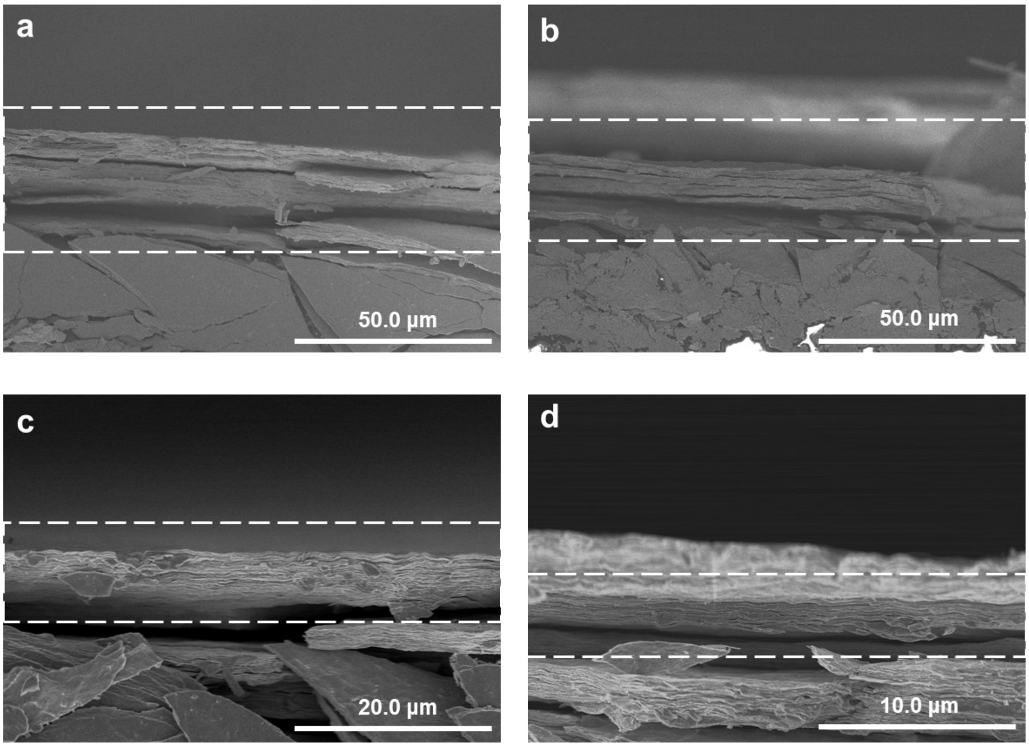

Fig. S9. Cross-sectional SEM images of hot-pressed $Ti_3C_2T_x$ MXene membrane with an initial thickness of ≈8.86 μm: (a) before and (b-d) after pressing with pressures of (b) 2, (c) 4, and (d) 6 MPa.

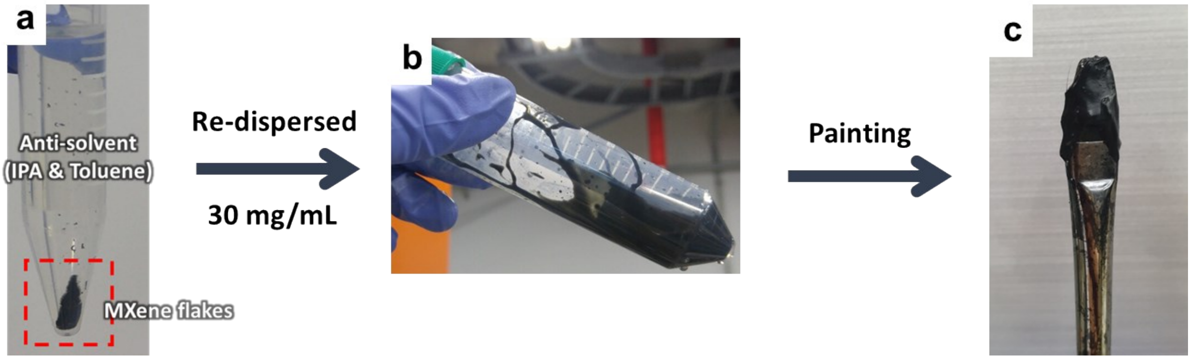

Fig. S10. (a) Sedimented $Ti_3C_2T_x$ MXene flakes by anti-solvents with a low polarity and under a centrifugation process. (b) Re-dispersed $Ti_3C_2T_x$ MXene flakes in DI water resulting in $Ti_3C_2T_x$-MXene ink with a high concentration and viscosity. (c) Painting with $Ti_3C_2T_x$ MXene ink using a brush.

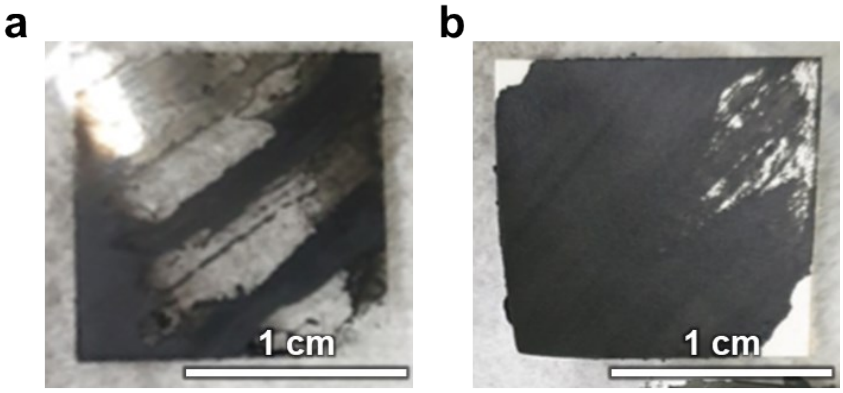

Fig. S11. Painted $Ti_3C_2T_x$ MXene ink with a concentration of 45 mg/ml on (a) glass and (b) filter paper.

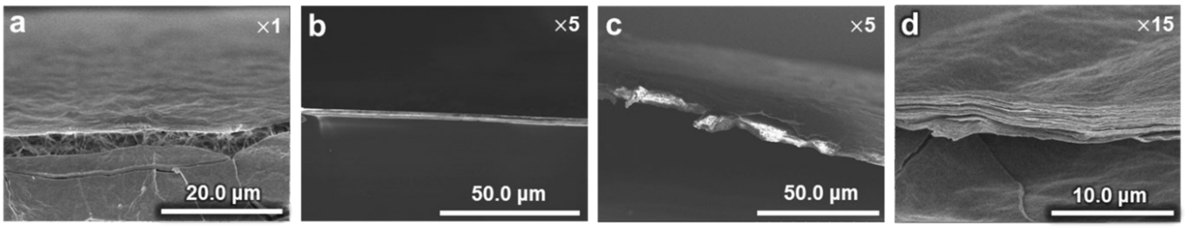

Fig. S12. SEM cross-sectional images of painted $Ti_3C_2T_x$ MXene ink on (a) PP filter, (b) glass, (c) PC filter, and (d) filter paper. The number of painting processes is included on the upper right in (a-d).

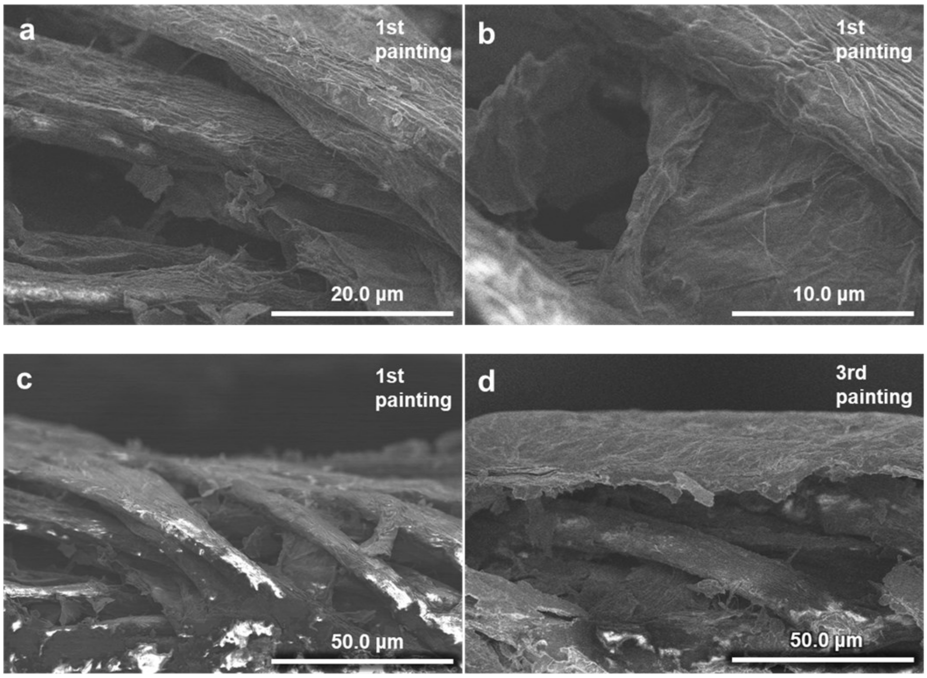

Fig. S13. SEM images of painted $Ti_3C_2T_x$ MXene ink on filter paper showing (a–c) coated $Ti_3C_2T_x$ flakes on fiber and (d) stacked $Ti_3C_2T_x$ flakes by subsequent painting.

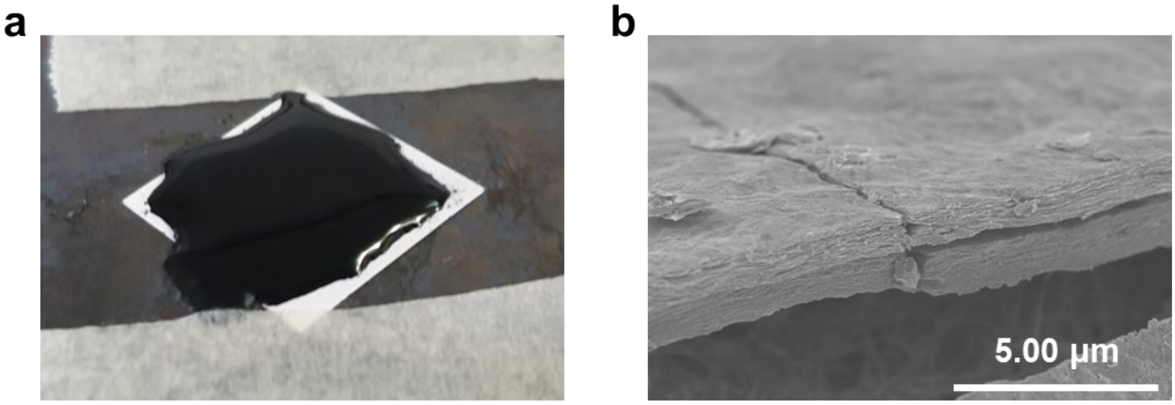

Fig. S14. (a) A photograph showing the aggregation and spills of painted $Ti_3C_2T_x$ MXene ink on hydrophobic PP filter. (b) SEM cross-sectional images of MXene ink painted five times on PP filter showing the fractured structure.

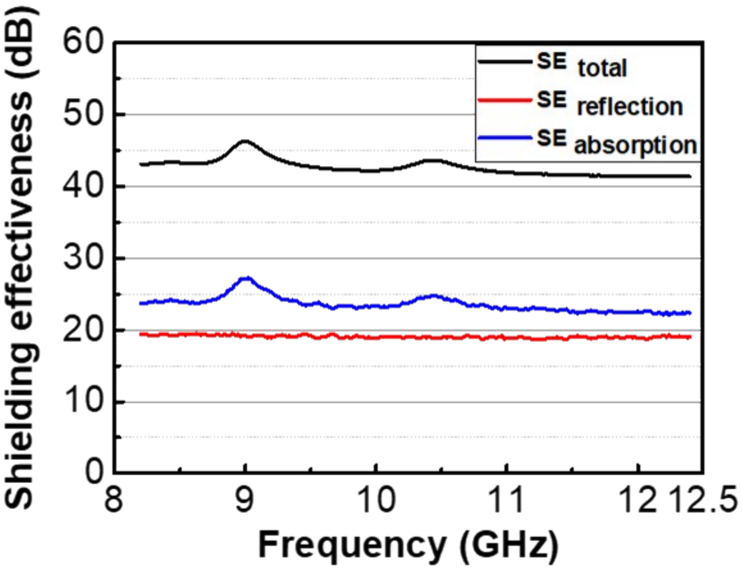

Fig. S15. EMI SE of pure MXene (thickness ≈13.6 μm)/PP membrane as a function of frequency.

Table S1. EMI shielding performance of various shielding materials.

Sample		Thickness (mm)	EMI SE (dB)	SE/*t* [a)] (dB/mm)	SSE/*t* [b)] (dB mm^2/g)**	Reference
Graphene-based	Graphene	0.0084	20	2381.0	1.1×10^6	48
	Graphene	0.3	46.3	154.3	6.8×10^4	49
	Carbon/graphene	0.073	51	698.6	9.7×10^5	50
	Graphene/PE	1	35	35.0	1.1×10^6	51
	CNT/graphene	1.6	38.4	24.0	4.1×10^6	52
	rGO/PS	2.5	32.4	13.0	1.4×10^5	53
	rGO/PDMS	3.4	30	8.8	1.5×10^5	54
	Graphene foam	3	37	12.3	1.8×10^5	55
CNT-based	MWCNT/SWCNT	0.13	65	500.0	6.1×10^5	56
	MWCNT/ABS	1.1	50	45.5	1.9×10^5	57
	SWCNT/PU	2	17	8.5	3.2×10^4	58
	CNT/cellulose	0.15	35	233.3	9.7×10^5	59
Metal foil	Al foil	0.008	66	8250.0	3.0×10^6	60
	Cu foil	0.01	70	7000.0	7.8×10^5	60
	CuNi	1.5	25	16.7	7.0×10^4	61
	SS/PP	3.1	48	15.5	2.4×10^4	62
MXene-based	$Ti_3C_2T_x$ foam	0.006	32	5333.3	1.4×10^7	63
	$Ti_3C_2T_x$ film	0.045	92	2044.4	8.5×10^5	60
	$Ti_3C_2T_x$/CNFs	0.074	26	351.4	2.2×10^5	64
	**Filtrated $Ti_3C_2T_x$**	**0.0136**	**46.3**	**3404.4**	**1.4×10^6**	**This work**
	**Painted $Ti_3C_2T_x$**	**0.0049**	**32.3**	**6591.8**	**2.8×10^6**	**This work**

[a)]SE/*t*: SE divided by sample thickness.
[b)]SSE/*t*: Specific SE, which is divided by the product of sample density and thickness.
